# Inherited Structure Properties of Larch Arabinogalactan Affected via the TEMPO/NaBr/NaOCl Oxidative System

**DOI:** 10.3390/polym16111458

**Published:** 2024-05-22

**Authors:** Vladislav A. Ionin, Yuriy N. Malyar, Valentina S. Borovkova, Dmitriy V. Zimonin, Roksana M. Gulieva, Olga Yu. Fetisova

**Affiliations:** 1 Institute of Chemistry and Chemical Technology, Krasnoyarsk Science Center, Siberian Branch Russian Academy of Sciences, Akademgorodok 50/24, Krasnoyarsk 660036, Russia; ionin.va@icct.krasn.ru (V.A.I.); bing0015@mail.ru (V.S.B.); zimonind89@mail.ru (D.V.Z.); roksanagu@yandex.ru (R.M.G.); fetisova@icct.ru (O.Y.F.); 2 School of Non-Ferrous Metals and Material Science, Siberian Federal University, Pr. Svobodny 79, Krasnoyarsk 660041, Russia

**Keywords:** larch, arabinogalactan, oxidation, 2,2,6,6-tetramethylpiperidin-1-oxyl (TEMPO), flocculation

## Abstract

Arabinogalactan (AG), extracted from larch wood, is a β-1,3-galactan backbone and β-1,6-galactan side chains with attached α-1-arabinofuranosyl and β-1-arabinopyranosyl residues. Although the structural characteristics of arabinogalactan II type have already been studied, its functionalization using 2,2,6,6-tetramethylpiperidin-1-oxyl (TEMPO) oxidation remains a promising avenue. In this study, the oxidation of AG, a neutral polysaccharide, was carried out using the TEMPO/NaBr/NaOCl system, resulting in polyuronides with improved functional properties. The oxidation of AG was controlled by analyzing portions of the reaction mixture using spectrophotometric and titration methods. To determine the effect of the TEMPO/NaBr/NaOCl system, air-dried samples of native and oxidized AG were studied by Fourier-transform infrared (FTIR) and nuclear magnetic resonance (NMR) spectroscopy, as well as by gel permeation chromatography. Compounds that model free (1,1-diphenyl-2-picrylhydrazyl (DPPH)) and hydroxyl radicals (iron(II) sulfate, hydrogen peroxide, and salicylic acid) were used to study the antioxidant properties. It was found that, in oxidized forms of AG, the content of carboxyl groups increases by 0.61 mmol compared to native AG. The transformation of oxidized AG into the H^+^ form using a strong acid cation exchanger leads to an increase in the number of active carboxyl groups to 0.76 mmol. Using FTIR spectroscopy, characteristic absorption bands (1742, 1639, and 1403 cm^−1^) were established, indicating the occurrence of oxidative processes with a subsequent reduction in the carboxyl group. The functionality of AG was also confirmed by gel permeation chromatography (GPC), which is reflected in an increase in molecular weights (up to 15,700 g/mol). A study of the antioxidant properties of the oxidized and protonated forms of AG show that the obtained antioxidant activity (AOA) values are generally characteristic of polyuronic acids. Therefore, the TEMPO oxidation of AG and other neutral polysaccharides can be considered a promising approach for obtaining compounds with the necessary controlled characteristics.

## 1. Introduction

**Figure 1:**
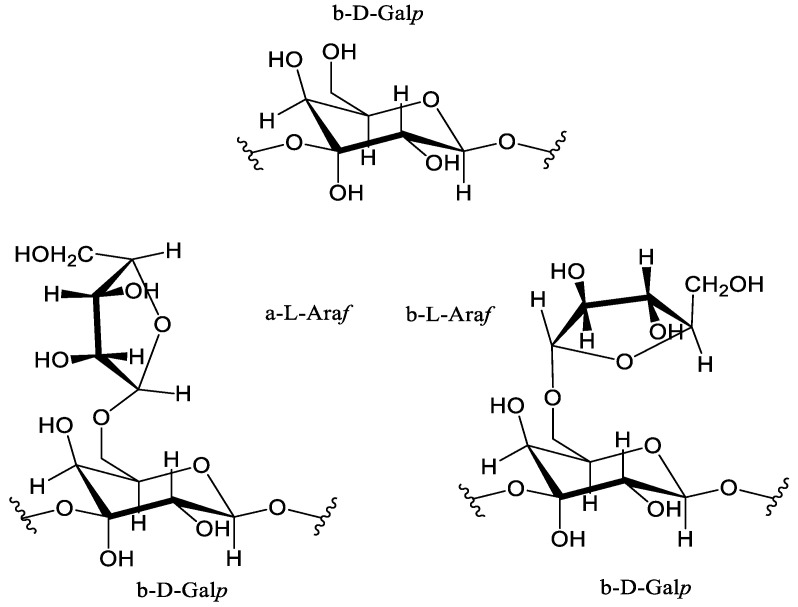
Structure of arabinogalactan units.

Among all of the polysaccharides present in the composition of larch hemicelluloses, isolated under relatively mild conditions [1], one of the most valuable is arabinogalactan, due to the prospects of its application in prebiotic additives [2,3], immunostimulators [2,4,5], so as the tumor cell metastases form inhibitors of its sulfated form [6]. Typically, the structure of AG obtained from plant biomass contains sequentially connected main units, represented by (1→3)-β-D-linked galactopyranoses with altered chains in the form of (1→6)-α-L- and (1→6)-β-L-linked arabinofuranoses [1] shown on Figure 1.

AG’s heterogeneous nature is relevant in the synthesis of biologically active drugs with specified properties [7,8], due to the high number of labile hydroxyl groups, easily affected by chemical interactions. Despite the significant advances made in arabinogalactan sulfating modifications [6,8], it still requires a more detailed study of the existing approaches to obtain reliably active AG-based drugs with further applications in the pharmaceutical and food industries [9].

One of the most promising directions for AG functionalization lies in carboxylation: high reactive carboxyl groups facilitate participate in their further conversion into ethers through alkylation, as well as the cross-coupling reaction [10,11]. Peroxides, periodates, hypochlorite, and ozone commonly used for biopolymer oxidation present disadvantages, including low selectivity processes, leading to byproduct formation due to side reactions [12,13]. In addition to possible radical polymerization reactions [14], hydrolysis takes place with the decomposition of bonds linking polysaccharide molecules’ elementary units, negatively affecting molecular weight and, as a consequence, the solubility and antioxidant activity of biopolymer-based drugs, which is extremely vital [11,15].

A perspective path due to its high regioselectivity and high reaction rate is the specific catalytic oxidation of the C-6 primary hydroxyl group of polysaccharides by the nitroxyl radical TEMPO, catalyzed by the interconversion of NaOCl and NaBr [16,17]. The success of the application of the above-mentioned techniques in modifying AG via TEMPO/NaBr/NaOCl-mediated oxidation is considered to be promising to obtain high-effective water-soluble derivatives [18,19,20], which can subsequently be used in the chemical, pharmaceutical, and food industries [21].

Moreover, due to its anionic nature, polysaccharide carboxyl groups are very effective in declining the charge density supplied by cations in aqueous solutions [22,23], which could possibly be used in operations connected with the removal of solid particles, colors, and dyes from wastewater by flocculation. Since raw-material polysaccharides are known to be biodegradable, harmless, and free from secondary pollution [3,24], they could potentially replace organic synthetic flocculants in the food and fermentation industries, when environmental impacts become a major concern [25,26].

Thus, this study focuses on the oxidation of arabinogalactan obtained from *Larix Sibirica Ledeb* larch wood via a TEMPO/NaBr/NaOCl medium, evaluating its impact on AG’s molecular weights examined by GPC methods, a process investigated by spectrophotometric carbazole and titration methods, while observing the structural changes in the obtained samples, which were registered by FTIR and NMR spectroscopy, with the determination of AOA using model-free and hydroxyl radical compounds. The flocculation ability of various amounts of oxidized AG regarding bentonite clay dispersion bridging with commonly used bivalent and trivalent cation salts (Ca^2+^ and Fe^3+^) is assessed to propose its further applications.

## 2. Materials and Methods

### 2.1. Raw Material

As a raw material for oxidation, air-dried arabinogalactan was used, isolated well as in the study [27], from Siberian larch wood (*Larix Sibirica Ledeb*) grown in the Krasnoyarsk Territory.

A total of 10 g of air-dried larch wood sawdust was loaded into a glass flask equipped with a reflux condenser and an overhead stirrer, with the addition of distilled water (liquid/wood ratio—15), followed by heating in a water bath for 5 h. The hot solution was separated from the wood by filtration with a Buchner funnel. The solution was concentrated on a rotary evaporator to a 1/5 volume. Arabinogalactan was isolated by precipitation with a fivefold volume of ethanol (96 wt. %) with slow stirring. The solution with the precipitate was kept in the refrigerator for 12 h, and then the isolated arabinogalactan was separated by filtration and then dried in a freeze-dryer: “Iney-6” (Iney, Moscow, Russian Federation).

The AG monosaccharide composition, produced according to method [28], revealed the presence of large amounts of galactose and arabinose at a ratio of 6:1 with small (less than 9 wt. %) amounts of arabinose, furfural, 5-HMF, and levulinic acid.

To perform flocculation tests, bentonite clay obtained from the Kaibalskoye-2 deposit (Bentonite of Khakassia LLC, Chernogorsk, Russian Federation) was used [29]. The quantitative composition was determined by X-ray diffraction using an X’pertPro diffractometer (PANalytical, Almelo, The Netherlands) with Cu radiation, a PIXcel detector, and a graphite monochromator. Rietveld refinement using Profex GUI was performed according to [30].

### 2.2. Larch Wood Arabinogalactan Oxidation via TEMPO/NaBr/NaOCl

**Figure 2:**
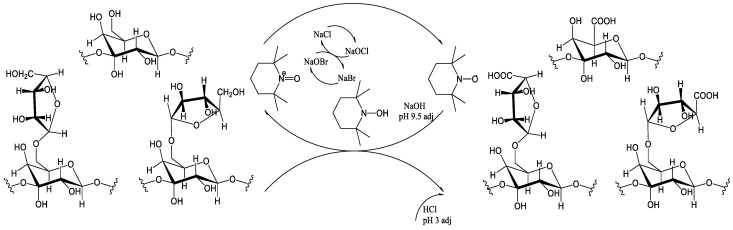
Scheme of TEMPO/NaBr/NaOCl-mediated oxidation of larch wood arabinogalactan.

The scheme of larch wood arabinogalactan TEMPO/NaBr/NaOCl-mediated oxidation is shown in Figure 2.

**Figure 3:**
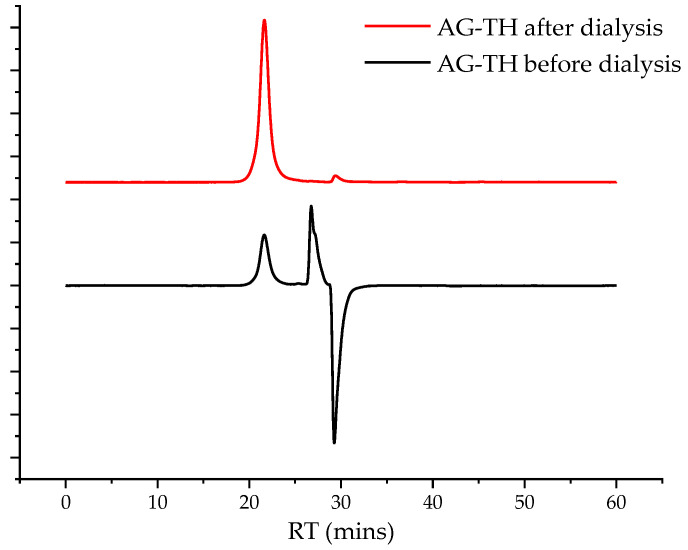
Gel permeation chromatograms of native and purified AG-T after dialysis.

The oxidation of arabinogalactan in the presence of the TEMPO/NaBr/NaOCl medium was performed using the techniques described in [31], with some modifications. In this study, 2 g of AG was dissolved in 500 mL of distilled water under stirring (400 rpm). After that, 0.8 g of NaBr and 0.04 g of TEMPO were added. The reaction started simultaneously with the gradual addition of 80 mL of a freshly prepared 15 wt. % NaOCl solution and adjusting the pH mixture with a 2 M HCl solution to 9.5. The reaction mixture was constantly cooled to a range of 2–5 °C in an ice bath and the pH of the reaction medium was maintained at 9.5 by adjusting it with a 0.1 M NaOH solution. The reaction was carried out until NaOH consumption stopped more than twice in a row. To reduce the carbonyl byproducts and terminate the reaction, 0.3 g of NaBH_4_ was added, the pH of the reaction medium was adjusted to 3 with the 2 M HCl solution, and 60 mL of ethanol with 0.06 g of NaCl was added and stirred for 45 min. The obtained aqueous solution was evaporated to 50 mL under vacuum conditions and further dialyzed using a 46 mm width cut-off dialysis bag, MF-5030-46 (MFPI, Seguin, TX, USA), with a 3.5 kg/mol pore size for 24 h in deionized water to eliminate low-molecular byproducts and salts, and was then transferred to a Petri dish and dried at 50 °C to reach a constant weight. A chromatogram showing the removal of low-molecular-weight impurities is preented in Figure 3.

The resulting dried, oxidized AG (AG-T) samples were converted to an acidic form (H^+^ form, AG-TH). This was maintained using a strong acid cation exchanger, KY-2-8 (PO “TOKEM”, Kemerovo, Russia), as described in [32], and then transferred to a Petri dish and dried at 50 °C to reach a constant weight.

The AG’s oxidation was controlled by analyzing the portions of reaction mixture using spectrophotometric and titration methods. The effects of oxidation system air-dried samples of native and oxidated AGs were studied by FTIR, NMR spectroscopy, and GPC. The AOA was studied using the compounds modeling free radical DPPH and hydroxyl radicals.

### 2.3. Arabinogalactan Hydrolysis and Spectrophotometric Determination of Formed Uronic Acids

The determination of uronic acids formed by AG’s strong acid hydrolysis was based on the carbazole method [33] using an Ecoview UV 6900 spectrophotometer (Shanghai Mapada Instruments Co., Ltd., Shanghai, China).

To create a calibration curve, a stock solution of analyzed uronic acid (0.0250 g of D-galacturonic acid (Sigma Aldrich, St. Louis, MI, USA) in 100 mL of distilled water) was diluted and the samples were hydrolyzed with concentrated sulfuric acid, as follows.

A 1 mL sample containing up to 200 μg of D-galacturonic acid was placed in a 10 mL test tube, then 6 mL of concentrated sulfuric acid was slowly added and cooled in an ice bath. After 0.5 mL of the 0.015 wt. % solution of carbazole–ethanol reagent was added, the reaction mixture was stirred for 30 s and incubated at 80 °C in the range of 5–10 min until a stable color change occurred. The tube was cooled to a room temperature, followed by a spectrophotometric measurement of the sample (λ = 530 nm), zeroed on distilled water as a reference solution.

**Figure 4:**
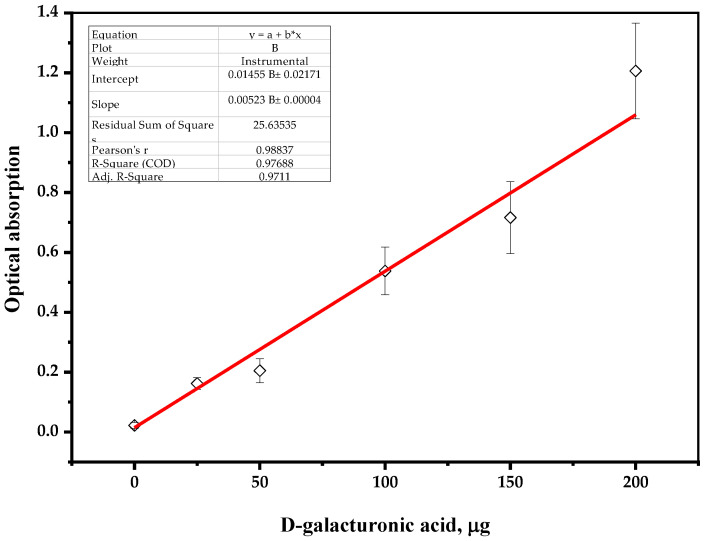
Calibration curve of D-galacturonic acid solutions.

The plotted calibration curve for the results of standard solutions of D-galacturonic acid is shown on Figure 4.

### 2.4. Determination of Native and Oxidated Arabinogalactan Carboxylic Contents with Potentiometric Titration and Its Impact on Solubility

The determination of carboxyl groups in AG samples was based on the electric conductivity titration method and was performed according to [34]: 0.3 g of AG sample diluted in 500 mL of distilled water and 20 mL of 0.1 M NaCl and 3 mL of 0.1 M HCl were added. After that, the mixture was dispersed continuously for 8 h at room temperature. Then, a 0.01 M NaOH solution was added at the rate of 0.1 mL/min up to pH 10 by using a pH stat. The carboxylate content of the sample was determined from the conductivity and pH curves.

To define native AG and its oxidized derivatives’ solubility, a dissolving test was performed: 0.1 g of each sample was dissolved in distilled water, slowly increasing the dilution at room temperature (25 °C) with long-term stirring using a laboratory vortex stirrer. The solubility of AG samples was determined by the amount of water used to completely dilute them according to the ASTM E1148-02 Standard Test Method for Measurements of Aqueous Solubility [35].

### 2.5. Fourier-Transform Infrared Spectroscopy

The AG samples’ IR spectra were registered using the Shimadzu IRTracer-100 FTIR spectrometer (Shimadzu, Kyoto, Japan) as follows: 2 mg of native or oxidized AG samples were pressed in tablets in a potassium bromide matrix.

### 2.6. Nuclear Magnetic Resonance Measurements

The AG samples’ ^1^H and ^13^C nuclear magnetic resonances were recorded using a Bruker Avance III 600 spectrometer (Bruker BioSpin, Rheinstetten, Germany). Before the analysis, the samples were completely dissolved in D_2_O and placed into a 5 mm NMR tube at room temperature.

### 2.7. Gel Permeation Chromatography

The weight average molecular weight, M_w_, and polydispersity index, PDI, of the AG samples were determined by GPC using an Agilent 1260 Infinity II GPC/SEC system (Agilent Technologies, Santa Clara, CA, USA) with a refractive detector and two Agilent PL Aquagel-OH Mixed-M columns with a 0.1 M NaNO_3_ solution combined with NaN_3_ in deionized water as a mobile phase. The calibration was performed using Agilent polyethylene glycol standards EasiVial PEG/PEO with M_p_ 106–500,000 g/mol (Agilent, Santa Clara, CA, USA). The eluent flow rate was 1 mL/min and the sample volume was 100 µL. Before the analysis, the samples were dissolved in a mobile phase (5 mg/mL) and filtered through a 0.45 µm Agilent PES membrane filter (Millipore, Burlington, MA, USA). Collected data were processed using Agilent GPC/SEC MDS software version 2.2.

### 2.8. Thermogravimetric Analysis

The TGA study was carried out on a NETZSCH STA 449 F1 Jupiter simultaneous thermal analysis instrument (NETZSCH STA 449 F1 Jupiter instrument, Netzsch, Selb, Germany). The AG samples were analyzed in argon at a heating rate of 10 °C · min^−1^, with temperatures ranging from ambient to 700 °C, with protective and blowout gas flow rates of 20 and 50 mL ∙ min^−1^, respectively. An Al_2_O_3_ cylindrical crucible with a perforated cover was used, and the reference was an empty corundum crucible with a cover. The instrument was calibrated according to the specifications using reference substances supplied by the instrument. The sample weight for the analysis was determined on a Sartorius BP121S analytical lab-scale digital balance. The measurement data were processed using NETZSCH. Proteus Thermal Analysis.5.1.0 software supplied by the instrument.

### 2.9. Antioxidant Activity

#### 2.9.1. DPPH Radical Scavenging Assay

The ability of AG, AG-T, and AG-TH to scavenge DPPH radicals was measured according to the method described in [29]. An aqueous AG solutions at various concentrations (0.2, 0.5, 1, and 2 mg/mL) reacted with 2 mL of a 0.2 mmol/L DPPH solution dissolved with anhydrous ethanol [24,29]. The mixture was well mixed and kept at room temperature for 30 min in the dark. After that, the absorbance was measured at 517 nm against a blank.

The calculation used to report the scavenging of DPPH radicals is presented Equation (1):

(1) $$\mathrm{DPPH}\;\mathrm{Radical}\;\mathrm{Scavenging}\;\mathrm{Ability}\;(\%)=\left(1-\frac{A_{S}-A_{B}}{A_{C}}\right)\;*\;100\%,$$

where A_C_ represents the absorbance of the DPPH solution without a sample, A_S_ is the absorbance of the test sample mixed with the DPPH solution, and A_B_ is the absorbance of the sample without the DPPH solution.

#### 2.9.2. Hydroxyl Radical Scavenging Assay

The ability of AG, AG-T, and AG-TH to scavenge hydroxyl radicals was determined as previously reported [36]. AG samples were dissolved in deionized water in concentrations of 0.2, 0.5, 1, and 2 mg/mL. The water sample solution (1 mL) was mixed with 1 mL of 6 mM ferrous sulfate and 1 mL of 6 mM H_2_O_2_. The mixture was evenly blended and incubated at 37 °C for 20 min. After that, 1 mL of 6 mM salicylic acid (dissolved with 50% *v*/*v* ethanol) was added, mixed thoroughly, and incubated for more 15 min at 37 °C The absorbance of the mixture was measured at 510 nm against the blank.

The capability to scavenge the hydroxyl radical was calculated using Equation (2):

(2) $$\mathrm{Hydroxyl}\;\mathrm{Radical}\;\mathrm{Scavenging}\;\mathrm{Ability}\;(\%)=\left(1-\frac{A_{S}-A_{B}}{A_{C}}\right)\;*\;100\%,$$

where A_C_ is the absorbance of the mixture without the sample, A_S_ is the absorbance of the test sample mixed with a reaction solution, and A_B_ is the absorbance of the sample without a salicylic acid solution.

### 2.10. Measurement of Flocculating Activity

To investigate the effect of AG-TH flocculation activity, experiments were performed based on polysaccharide’s interaction with suspended bentonite supplemented with metal ions, including CaCl_2_ and FeCl_3_, using modified techniques [22,25] as follows.

The flocculating mixture was obtained using 5 mL of a 10 g/L bentonite clay suspension pretreated with an ultrasonic homogenizer (Volna-M, Moscow, Russian) and adjusted to pH 7. A total of 0.2 mL of a 0.04 M metal salts aqueous solution and a range of 1–5 mL of a 0.1 g/L AG-TH solution were used to estimate the effect of polysaccharide flocculating activity. The reaction mixture was stirred using a Vortex mixer for 30 s and then kept still for 5 min. The absorbance of the supernatant and the AG-TH-free blank control was measured using an Ecoview UV 6900 spectrophotometer (Shanghai Mapada Instruments Co., Ltd., Shanghai, China) at 550 nm (as OD550 and OD550, respectively). All assays were conducted in triplicates. The flocculating rate and activity were calculated using Equations (3) and (4):

(3) $$\mathrm{AG-TH}\;\mathrm{Flocculating}\;\mathrm{Rate}\;(\%)=\left(\frac{OD550-OD550,blank}{OD550}\right)\;*\;100\%,$$

(4) $$\mathrm{AG-TH}\;\mathrm{Flocculating}\;\mathrm{Activity}=\left(\frac{1}{OD550}\right)-\left(\frac{1}{OD550,blank}\right).$$

## 3. Results

### 3.1. Arabinogalactan Oxidation Dynamics and Yields of Its Oxidized Derivatives

As mentioned above, AG, isolated from plant biomass, is represented by sequentially chained elementary units of galactopyranoses and arabinofuranoses. It is assumed that, as the TEMPO/NaBr/NaOCl-mediated reaction provokes the hydroxyl groups’ conversion into a carboxylic form, it leads to a pH decrease in the reaction medium, which must be maintained in the range of 9.5–10.0 [16,17] with a 0.1 M solution of NaOH. The NaOH consumption rate interference depends on nitroxyl radical aggregation by NaOH-NaOCl-NaBr conversion, so as to forma AG’s carboxylates.

**Figure 5:**
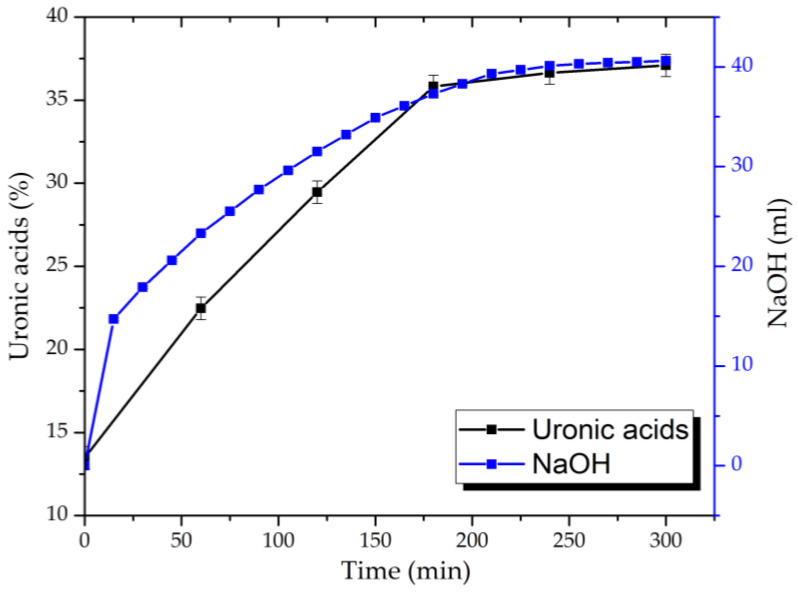
Dynamics of 0.1 M NaOH consumption and uronic acid content.

Based on the consumption rate of NaOH during the process and the data obtained for uronic acid content in hydrolysates of the reaction medium determined by the carbazole–ethanol method, the dynamics of the oxidation are established (Figure 5).

It has been demonstrated that, during the AG’s oxidation via the TEMPO/NaBr/NaOCl system, the 0.1 M NaOH solution’s consumption process by the reaction mixture takes place in the first 60 min, with insignificant changes after 240 min. On the other hand, uronic acid content in the reaction-medium hydrolysates rises more than 2.7 and reaches the maxima from 13.4 ± 0.7 to 37.1 ± 0.7% in 180 min with no visible difference.

Since the above-mentioned carbazole method is based on AG sulfuric acid hydrolysis with the determination of uronic acid by the D-galacturonic acid analytical standard solution, it could possibly overlook the carboxylic groups formed by arabinofuranoses oxidation. In addition, AG hydrolysis in the sulfuric acid medium is an unstable process, leading to molecular-chain decomposition with the subsequent oxidation of its elementary units that are not involved in the conversion process via the TEMPO/NaBr/NaOCl system.

The resulting yields of oxidized AG samples with their following dialysis and conversion into the H^+^ form (AG-TH) vary at 76.27 ± 6.25 wt. %.

### 3.2. Carboxylic Acid Group Content and Its Impact on Solubility

**Figure 6:**
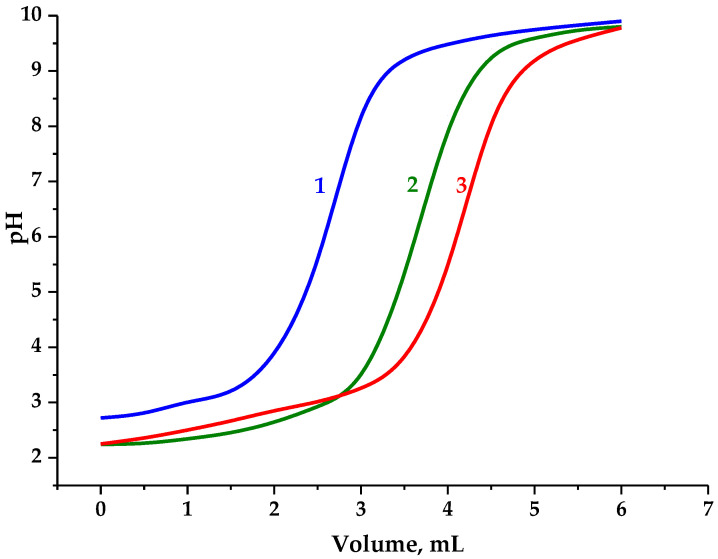
Potentiometric titration curves of arabinogalactan samples: (**1**) native AG; (**2**) AG-T; (**3**) AG-TH.

Therefore, to precisely assess carboxyl group content, this was determined by the potentiometric titration of the AG samples represented in Figure 6.

Since native arabinogalactan contains an insignificant amount of carboxyl groups, the consumed volume of the NaOH solution during its titration to pH 7 was considered to neutralize the hydrochloric acid used in the analyzed solution, which was subsequently used to evaluate the oxidation effect.

The content of carboxyl groups was determined, according to Dalton’s law of interaction of a monobasic acid with an alkali at a ratio of 1 to 1, taking into account the volume used for native AG titration.

It has been established that AG oxidation via TEMPO/NaBr/NaOCl and the adjustment to pH 3 increase the carboxyl group content (0.61 mmol) compared to the native AG. Taking into account the proportion of elementary units containing hydroxyl groups capable of monobasic acids promotion, the content of carboxyl groups compared to native AG rises to 34.5 wt. %. The conversion of AG-T to the H^+^ form (AG-TH) using a strong acid cation exchanger leads to an increase in active carboxyl groups to 0.76 mmol or 43.1 wt. % compared to native AG.

Hence, the results obtained correlate with the above-mentioned data of uronic acid content in hydrolysates of the AG oxidation medium adjusted due to the presence of arabinofuranoses.

**Figure 7:**
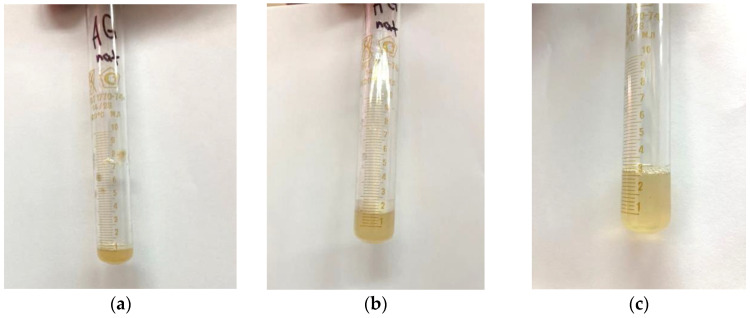
Dissolving the native arabinogalactan (**a**) 100 mg/mL; (**b**) 50 mg/mL; (**c**) 33 mg/mL.

After a visual assessment, it was found that the solution had a cloudy yellow color with small undissolved particles (Figure 7a).

The addition of distilled water to 2 mL led to the complete dissolution of the particles in the solution (Figure 7b), and a further increase in the volume of the solvent led to a clear solution (Figure 7c), which indicates that the approximate solubility of the native arabinogalactan is ~50 mg/mL.

**Figure 8:**
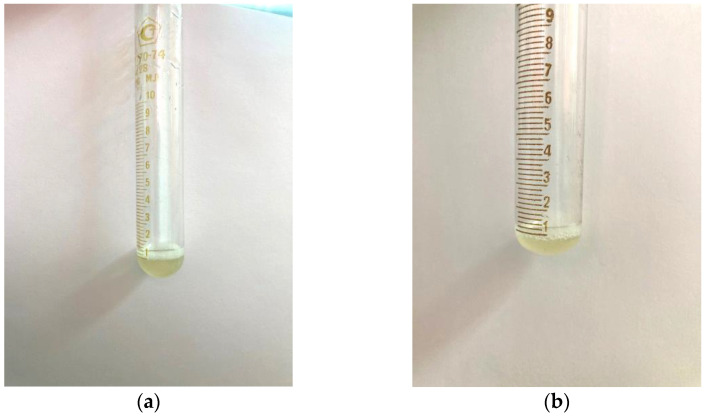
Dissolving the arabinogalactan samples up to a 100 mg/mL concentration (**a**) AG-T; (**b**) AG-TH.

A similar test was carried out with samples of AG-T and AG-TH. It was established that, as a result of the modification of the structure of the native AG, its solubility in water under the same conditions increased. Already, with the addition of 1 mL of distilled water, the modified samples completely dissolved, and the solutions had a more transparent appearance (Figure 8a,b).

**Figure 9:**
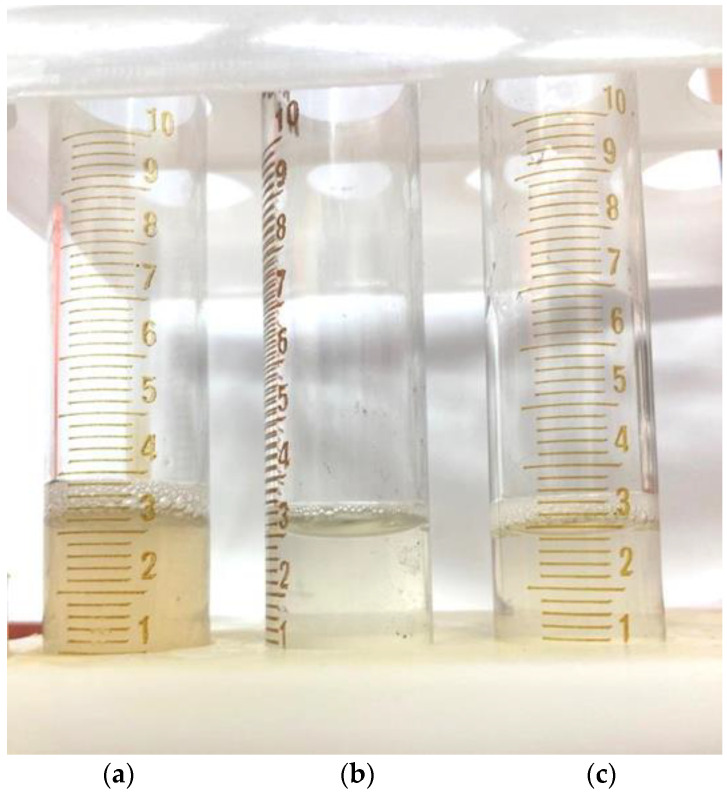
Dissolving the arabinogalactan samples up to a 33 mg/mL concentration: (**a**) native AG; (**b**) AG-T; (**c**) AG-TH.

Further dissolution leads to the complete discoloration of these solutions compared to the native AG solution (Figure 9).

### 3.3. Fourier-Transform Infrared Spectroscopy

**Figure 10:**
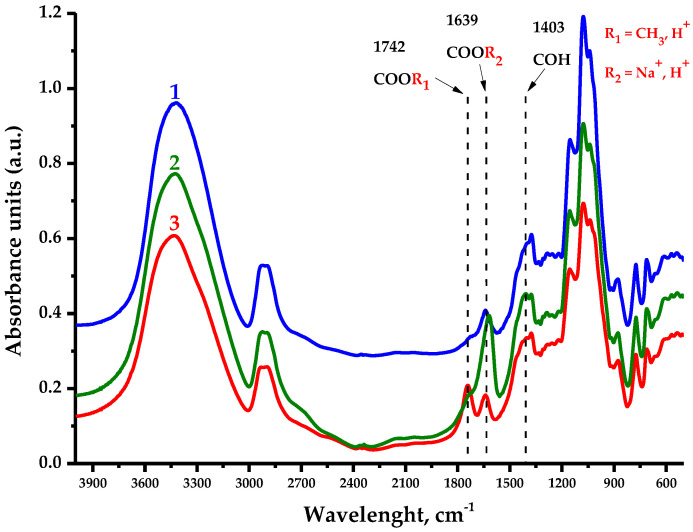
IR spectra of arabinogalactan samples’ absorbance units: (**1**) native AG; (**2**) AG-T; (**3**) AG-TH.

The FTIR spectra of the AG samples presented in Figure 10 contain all the bands characterizing the polysaccharides, in which the following features could be distinguished.

The large absorption unit (a.u.) observed in the native and oxidized samples’ spectra in the 3400 cm^−1^ region is associated with O-H stretching vibrations, while the a.u. in the 2900 cm^−1^ region characterize the stretching vibrations of primary alcohols’ aliphatic CH_2_ groups [37]. The observed a.u. redistribution in the 1742 cm^−1^ region has the most significance in this study and corresponds to the vibrations of methylesterified carboxyl groups [38], outstanding for AG-TH. Meanwhile, the a.u. in the 1639 cm^−1^ region is associated with the substituted carboxylate anion form, as well as intra- and intermolecular hydrogen bond formations of bounded water [37,39], which are more typical for AG-T. The fingerprint region of a.u. at 1403 cm^−1^ corresponds to the C–OH deformation vibration with the O–C–O symmetric stretching vibration due to uronic acid’s high carboxylate group contribution [39].

A.u. in the 1310–1220 cm^−1^ region could be attributed to multiple vibration models to characterize C-H-containing compounds; nevertheless, the most likely are the methyl component C-H planar deformation vibrations of acetylated functional groups [37,40]. Also, a.u. in the 1150–1040 cm^−1^ region indicates the presence of C-O-C glycosidic bonds’ stretching vibrations, inherent to polysaccharide units [41], while a.u. values at 880 cm^−1^ and 775 cm^−1^ correspond to the stretching vibration signals characterizing the nature of β-galactopyranose and arabinofuranose links, respectively [37,42,43]. It is common for a.u. values for AG and AG-T to also experience a mild oxidation of hydroxyl groups with С-О-С bonds [37] linking the polysaccharide units.

### 3.4. Nuclear Magnetic Resonance Measurements

In the course of AG oxidation via TEMPO/NaBr/NaOCl, there is a possible byproduct formation as a result of ongoing esterification reactions. In general, this is initiated by the nucleophilic attack on the TEMPO carbonyl form (or another low-molecular compound splintered off the polymer during the process) by hydroxyl groups abundantly present in AG leading to the formation of esters. To prevent these reactions in the medium after oxidation, the reduction in carbonyl groups by NaBH_4_ was performed, followed by dialysis to cut off low-molecular compounds, which also contaminate desirable oxidized AG-T (Figure 3) with its conversion into an H^+^ form (AG-TH) using a strong acid exchanger.

**Figure 11:**
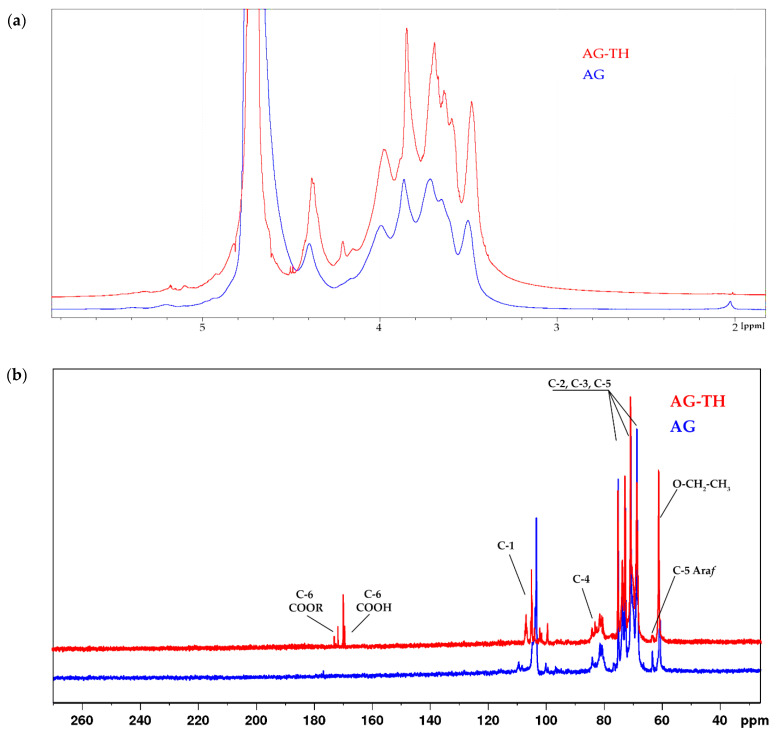
^1^H (**a**) and ^13^C (**b**) NMR spectra of native and oxidized AG recorded in D_2_O.

To establish the most common functional groups for native and oxidized AG-TH, ^1^H NMR spectra of native and oxidized AGs were recorded in D_2_O, shown on Figure 11a.

It has been supposed that the 3.7 ppm signal refers to the esterified groups of galacturonic acid units substituted by hydrogen atoms or their cationic form, which is noticeably different for the AG-TH sample caused by cationic dialysis. Moreover, the difference between the native and oxidized samples is the presence of a 2.02 ppm signal in native AG, which correlates to small numbers of methoxyl groups (-COCH_3_), which was also observed in AG monosaccharide structures [44].

In addition, the other characteristic signals of oxidized AG forming galacturonic acid units were assigned to corresponding protons (H-2, 3.62 ppm; H-3, 3.96 ppm; H-4, 4.16–4.21 ppm, and H-5, 4.37 ppm). The H-1 hydrogen atom, which is also present in the galacturonic unit, refers to signals in the range of 4.9–5.1 ppm. Such a scatter may be due to the difference in the main and side-chain substituents of arabinogalactan.

The ^1^H NMR data are consistent with the ^13^C NMR. In the low-field region of oxidized AG, typical signals of the C-6 carboxyl group of galacturonic acid units are observed at δ 173 and 174 ppm (esterified) and δ 170 ppm (non-esterified) [45]. In the anomeric region, overlapping signals at δ 100–108 ppm correspond to the C-1 galactose and arabinofuranose units in native AG, and after modification, galacturonic acid signals are added to the AG-TH spectrum (Figure 11b).

In the region of δ 81-85 ppm, there are signals related to C-4 atoms, and the set of signals corresponds to C-2, C-3, and C-5 of all types of units of both native and oxidized AG. Separately, at δ 63.8 ppm, there is a characteristic signal (C-5) of arabinofuranose [46]. The signal at δ 60.1 ppm probably corresponds to the ethyl ester group, which can be formed by the interaction of the carboxyl group of oxidized AG with ethyl alcohol during the reaction.

### 3.5. Gel Permeation Chromatography Study of Arabinogalactanes

**Figure 12:**
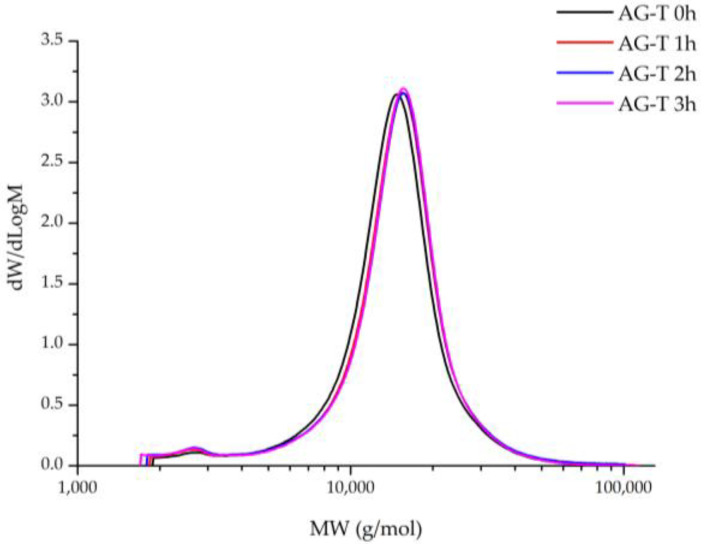
Molecular weight distribution of arabinogalactan samples.

The polymer molecular weight distribution (MWD) revelation during chemical modification processes helps to achieve and precisely evaluate the reaction’s progress, as well as the appropriate changes in the polymer’s structure, including byproduct formation, to prevent it in future. In this work, the MWD of TEMPO/NaBr/NaOCl-mediated AG oxidation products was determined depending on the process duration (Figure 12).

It has been observed that the MWD profiles of both the native and oxidated AG products were monomodal with a low polydispersity index. Native AG is characterized by a narrow MWD at M_p_ 14,876 g/mol and a polydispersity index of 1.27 (Table 1), reaching its maxima, 15,659 g/mol and 1.34, respectively, at the 3rd hour of the TEMPO/NaBr/NaOCl-mediated oxidation.

It is supposed that an increase in the polymer molecular weight is affected directly by the carboxyl groups’ formation. Noticeably, the difference between the M_p_ values of samples at 2 and 3 h is insignificant, which correlates with the data obtained for alkali consumption and uronic acid content during TEMPO/NaBr/NaOCl-mediated oxidation (Figure 5). Moreover, it obviously indicates the limit of AG structure saturation by carboxylic groups has been reached with the perseverance of the MWD profiles for all samples. Nevertheless, it was observed that AG-T 1 h MWD curves overlap by AG-T 2 h, but differ insignificantly. Unaltered low-molecular (less than 5000 g/mol) regions of oxidized AG indicate the absence of side processes, glycosidic bond hydrolysis of both the side and main chains of the polysaccharide, in particular.

### 3.6. Thermogravimetric Analysis

**Figure 13:**
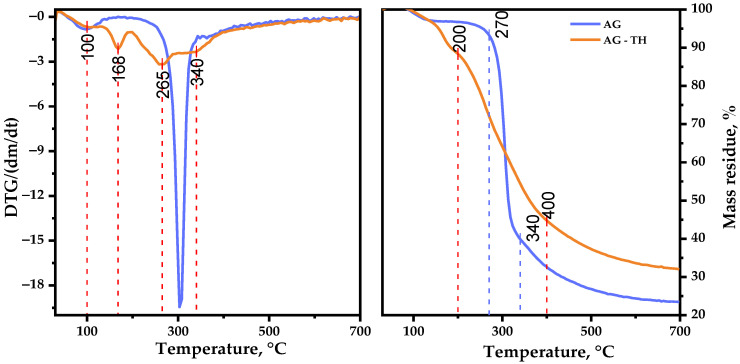
TGA/DTG thermal degradation profiles of native and oxidized AG samples.

It is well known that thermal decomposition behavior depending on the heterogeneous nature of underlying chemical reactions proceeding, due to simultaneous bond degradation, encourages changes in molecule conformation and phase conversion, which is crucial for biopolymer-obtained materials to determine their application [47]. Figure 13 represents the TGA/DTG curves recorded for AG and AG-TH in the range of 30–700 °C, where the following common and different features of thermal events should be mentioned.

The first stage (approximately at 95 °C) initiates with the evaporation of sorbed and crystallized moisture by AG samples [48], which typically leads to insignificant changes in weight loss (2.8 wt. %). A further increase in the temperature up to 250 °C has almost no effect on the native AG structure, which could be explained by the hydrogen bond decay of polar functional groups of polysaccharides.

The main, native, AG decomposition proceeds at the second stage of thermolysis: the sample intensively degrades at 300 °C with a maxima mass loss (55.0 wt. %). At the same time, the AG-TH sample’s weight loss initiates at lower temperatures and occurs at a slightly wider temperature range (130–400 °C) resulting in 52.2 wt. %. The DTG curve of the oxidized sample shows two distinct peaks, with maxima at 168 and 265 °C. In the course of the present study, it was supposed that AG and its oxidized derivative weight loss occurred through glycosidic bond decay, with some violation [49] followed by monosaccharide components’ depolymerization of AG.

The third stage of native AG decomposition (335–700 °C) is characterized by less weight loss (16.7 wt. %), which corresponds to AG carbonization processes, leading to the formation of coke residue [50], resulting in 23.5 wt. % mass residue at the end of the thermolysis. Oxidized AG-TH sample degradation differs with the temperature range (400–700 °C), with a mass residue of 32.0 wt. % at the end of decomposition.

Based on the obtained data, the activation energy of the samples’ thermal decomposition main stages was estimated. The AG thermal decomposition activation energy in the range of 250–335 °C amounts to 140.4 kJ/mol. At the same time, AG-TH activation energy at an active decomposition range (130–400 °C) is characterized by lesser values: 22.3 kJ/mol.

This AG-TH thermal decomposition behavior may be the consequence of disordered regions caused by ester group formation [51]. As a result, the degradation process of AG-TH initiates earlier at lower temperatures compared to AG, but the ordered regions of oxidized polysaccharide resist decomposition, which leads to higher thermal stability with sample mass perseverance.

### 3.7. Antioxidant Activity

Plant polysaccharides and their functionalized derivatives have great potential for applications in the pharmaceutical and food industries. One of the most high-valued properties is the ability to inhibit active radicals, which is commonly referred to as AOA [24].

To estimate the AOAs of AG-T and AG-TH, an analysis based on DPPH and hydroxyl radical scavenging was performed. These results are often expressed as IC_50_ concentration values (mg/mL) (Table 2), i.e., polysaccharides evincing antioxidant properties are considered to reduce radicals by 50% in terms of their effectiveness [24,52].

It has been observed that the determined IC_50_ values for the AG-T sample based on both DPPH and hydroxyl radical scavenging (68.5 and 2.1 mg/mL, respectively) differ for AG-TH (15.1 and 2.7 mg/mL, respectively) without visible patterns. It is not entirely correct to state that one of the AG samples exhibited a better AOA performance compared to another.

**Figure 14:**
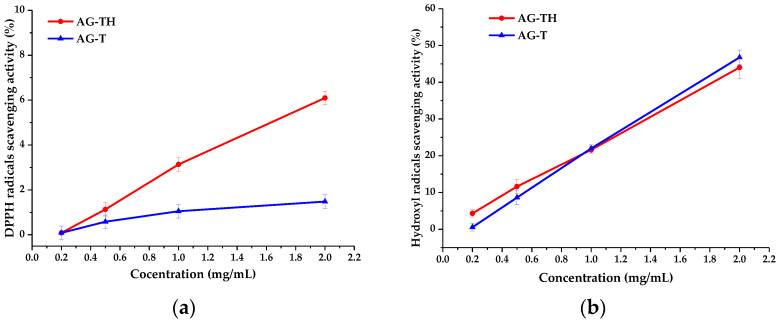
Activity of arabinogalactan samples scavenging (**a**) DPPH and (**b**) hydroxyl radicals.

Still, the protonated form of AG has a better ability to inhibit DPPH radicals (Figure 14a), which is related to the significantly higher mobility of H^+^ ions compared to Na^+^ ions in the AG-T sample. However, the differences in hydroxyl radical inhibition (Figure 14b) are insignificant and present an experimental error.

The obtained AOA values are common for polyuronic acids in which IC_50_ DPPH values reach more than 17 [52], while the inhibition of a more labile hydroxyl radical could not exceed 2 [52,53].

### 3.8. AG-TH Concentrations’ Effect on Flocculating Capability

It has been assumed that the obtained AG-TH samples participate in various interactions with bivalent and trivalent ions of metals, such as flocculation [22,25,26], due to its anionic nature. In general, such flocculation agents cause the aggregation of cells and particles of pollutants by bridging with cation charges.

**Figure 15:**
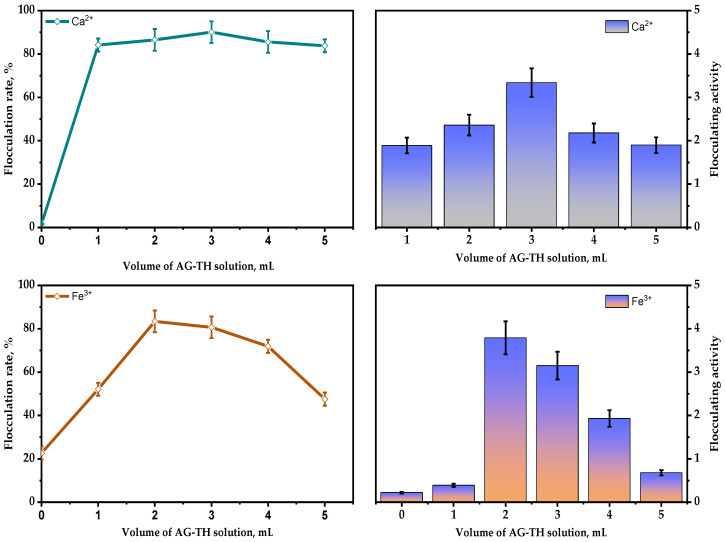
Impact of AG-TH volume on flocculation rate and flocculation activity in the presence of CaCl_2_ and FeCl_3_.

Therefore, flocculating activity was determined using the same amounts of Ca^2+^ and Fe^3+^ (shown on Figure 15) salts as the most commonly used component to stimulate the polysaccharides’ flocculation capability. This was performed using bentonite clay dispersion (10 g/L), which was based on XRD data with Rietveld refinement, mainly composed of montmorillonite—74.4 ± 0.6 wt. %, quartz—20.6 ± 0.4 wt. %, kaolin—3.3 ± 0.3 wt. %, anorthite—0.8 ± 0.1 wt. %, and anatase—0.9 ± 0.1 wt. %.

As it indicated above, it is clear that, as a result of the rising quantities of AG-TH (0.01–0.05 g/mL), in bentonite dispersion, the bivalent Ca^2+^ ions still promote flocculating activity and rate with maxima at 3.3 and 90.1%, respectively. It has been established that, in the absence of AG-TH, Ca^2+^ ions affect bentonite particles’ aggregation insignificantly, which results in zero flocculation capability, which also could be interpreted as sedimentation processes of bentonite.

In contrast to bivalent cations, the appropriate amount of Fe^3+^ ions in the absence of AG-TH still could enhance the flocculation rate, reaching up to 22.6%, with flocculation activity at a 0.22 level, which is used to amend additional data. Nevertheless, AG-TH presence (0.02 g/mL) leads to a maxima of flocculation activity and rate at 3.79 and 83.4%, respectively.

The obtained results indicate that, in presence of the same bivalent and trivalent cation concentrations, the optimal amounts of AG-TH (0.02–0.03 g/mL) enhance the flocculating capability, with an increase depending on the valence number of cations used. This could also be possible due to changes in charge densities, which were previously observed in accordance with Schulze Hardy’s law [25]. The above-mentioned results demonstrate the efficiency of AG-TH-mediated flocculation processes in high-concentrated bentonite dispersions, which could be possibly used for wastewater purification.

## 4. Conclusions

In this study, AG oxidation was performed via the TEMPO/NaBr/NaOCl system, followed by cut-off dialysis and a strong acid exchanger to obtain a purified product. The impact of the used oxidation system on byproduct formation, the amount of carboxyl groups, molecular weight, terminal structures, and antioxidant and flocculation capabilities were systematically investigated by spectrophotometric and titration methods. According to these methods, the oxidation outcome could be estimated, so as to determine the oxidation pattern of AG via the TEMPO/NaBr/NaOCl system.

It was observed that the AG oxidation proceeds in a mild way, with the perseverance of glycoside bonds linking polysaccharide units, mainly affecting hydroxyl groups, which corresponds with the data obtained by FTIR, NMR, and GPC methods. Based on the TGA/DTG curves, it has been demonstrated that oxidized AG-TH exhibits higher thermal stability compared to native AG due to the modification process. Nevertheless, it was observed that the degradation of AG-TH initiates at lower temperatures compared to the AG sample, which could be explained by the large number of ester bonds in the structure as the result of the oxidative modification process.

The evaluation of one of the most prospective applications oxidized AG was performed. The flocculating capabilities revealed that even a low concentration of AG-TH samples adjusts the flocculation rate of bentonite clay bridging bivalent and trivalent cations commonly used. Nevertheless, AG’s antioxidant activity values are generally characteristic of polyuronic acids, witnessing a perseverance of inherited properties of native AG. The obtained results suggest that AG oxidated via TEMPO/NaBr/NaOCl has a promising potential in processes that require solid–liquid separation and aggregation control. However, further research is warranted in modifying formed carboxyl groups to obtain highly effective water-soluble AG derivatives, which subsequently could be used in the chemical, pharmaceutical, and food industries.

## Figures and Tables

**Table 1:** Molecular weight averages of AG samples.

Samples	M_p_ (g/mol)	M_n_ (g/mol)	M_w_ (g/mol)	PD
AG-T 0 h	14,876	11,993	15,248	1.27
AG-T 1 h	15,526	12,135	15,846	1.31
AG-T 2 h	15,659	11,923	15,775	1.32
AG-T 3 h	15,659	11,984	16,001	1.34

**Table 2:** AG samples’ IC_50_ values in relation to DPPH and hydroxyl radical scavenging.

Samples	IC_50_ Values (mg/mL)	
	DPPH	•OH^−^
AG-T	68.5	2.1
AG-TH	15.1	2.7

## Data Availability

All data generated during this study are included in the article.
